# Frozen Elephant Trunk With Terumo Hybrid Plexus Prosthesis: A French Postmarket Longitudinal Study With Midterm Results

**DOI:** 10.1016/j.atssr.2025.07.024

**Published:** 2025-08-28

**Authors:** Thierry Caus, Yuthiline Chabry, Arvind Appa, Vito Giovanni Ruggieri, Marc Villaret, Bertrand Marcheix, Paul Achouh, Fabien Koskas

**Affiliations:** 1 Department of Cardiac Surgery, University Hospital and Université Picardie Jules Verne, Amiens, France; 2 Department of Cardiac Surgery, University Hospital Henri Mondor, Créteil, France; 3 Department of Cardiovascular and Thoracic Surgery, Felix Guyon University Hospital, Saint-Denis, Reunion, France; 4 Department of Thoracic and Cardiovascular Surgery, University Hospital Cabrol, Reims, France; 5 Terumo Inc, Guyancourt, France; 6 Department of Cardiac Surgery, University Hospital Rangueil, Toulouse, France; 7 Department of Cardiac Surgery, HEGP and Université Paris Cité, Paris, France; 8 Department of Vascular Surgery, Pitié-Salpetrière, Paris, France

## Abstract

**Background:**

We report on midterm follow-up of the EPI-Flex postmarket study in France, assessing the safety of the Thoraflex on a nationwide scale.

**Methods:**

A prospective, multicenter trial was conducted of all frozen elephant trunk procedures using Thoraflex in France between April 4, 2016, and April 3, 2019. Patients were divided into elective surgery (n = 214) and nonelective surgery (n = 137). We defined safety end points as age-adjusted 3-year survival including in-hospital mortality and a composite secondary end point including new stroke, spinal cord injury, acute kidney injury requiring dialysis, recurrent laryngeal nerve palsy, massive blood transfusion (>10 packs of red blood cells), and unexpected repeated thoracic endovascular aortic repair or aortic surgery within 30 days.

**Results:**

We included 351 patients (73% male; median age, 61 years; interquartile range, 55-70 years). In-hospital mortality rate was 54 of 351 (15.4%) and did not differ between elective and nonelective cases (*P* = .1). Classification random forest analysis, trained for 16 preoperative and perioperative covariates with 500 trees, identified age, deep hypothermia, and each time of cardiopulmonary bypass, visceral ischemia, or myocardial ischemia as the most influential factors associated with in-hospital mortality. Including in-hospital mortality, 3-year age-adjusted survival rates for elective and nonelective patients were 76% ± 6% and 70% ± 8%, respectively (*P* = .23). The composite end point significantly affected in-hospital mortality (*P* = 10^−5^) and 3-year age-adjusted survival rates (*P* = 10^−3^).

**Conclusions:**

Our results highlight that frozen elephant trunk with a Thoraflex remains a complex and evolving strategy for aortic arch diseases. Careful patient selection and optimized procedure engineering are essential to mitigate procedural risks.

## Visual Abstract

**Figure:**
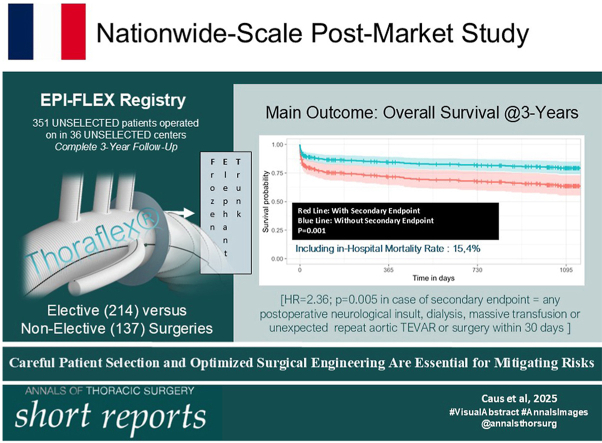

**In Short**

▪ A prospective multicenter national analysis was conducted of outcomes after a 3-year follow-up of 351 frozen elephant trunks with a Thoraflex in France.
▪ Assessment of risk factors of in-hospital mortality with machine learning algorithms underlines the importance of patient selection (age) and of time of visceral ischemia over timing of surgery or center volume.
▪ New stroke, spinal cord injury, dialysis, and major transfusion significantly worsen early and late survival (hazard ratio, 2.34).

The recent scientific joint societies guidelines^1^ highlight the usefulness of new hybrid prostheses in frozen elephant trunk (FET) procedures to treat aortic arch and proximal descending aorta. Controversies still exist, especially about technical challenges and potential risks, therefore questioning widespread use by nonexpert centers. Considering this background, we report on the French experience with the Thoraflex (Terumo Aortic) through the EPI-Flex trial, which we designed to capture all cases performed on a nationwide scale during the study period and focused on device safety.

## Material and Methods

### Study Design and Patients

The EPI-Flex (ClinicalTrials.gov identifier: NCT03735472) is a single-arm, prospective, and multicenter trial. As part of postmarket approval, EPI-Flex reports on all recipient patients operated on in France between April 4, 2016, and April 3, 2019. The study received ethical approval from Confidentiality of Clinical, Technical, and Research Information Systems on July 16, 2015 (No. 15.546).

### Surgical Procedure

This study reports almost exclusively (344/351) on the use of the Terumo Hybrid Plexus prosthesis. Typically, surgeons chose to perform separate head vessel reconstruction either as a second step immediately after the distal aortic anastomosis or as a third step following both distal and proximal aortic anastomosis to reduce myocardial ischemia. No patient underwent a head vessels–first approach. For most patients, the collar of the prosthesis was sewn in zone 2 or 3, as described in the original technique.

### Safety Assessment

Age-adjusted 3-year mortality, including any death during index hospitalization, was the main end point of the trial. We also defined a composite secondary end point as the percentage of patients presenting with any of the following complications: new stroke, spinal cord injury, acute kidney injury requiring dialysis, recurrent laryngeal nerve palsy, massive blood transfusion (>10 packs of red blood cells), and unexpected repeated thoracic endovascular aortic repair or aortic surgery within 30 days.

### Complications

An independent Clinical Events Committee based on patient-level source documents, obtained from the participating centers, adjudicated all complications.

### Study Population

We included all eligible FET procedures using a Thoraflex between April 4, 2016, and April 3, 2019 (Supplemental Figure 1). We divided patients between elective and nonelective FET procedures to treat aortic dissections (acute, subacute, or chronic), aneurysms, and other less common diseases like penetrating atherosclerotic ulcers, intramural hematoma, false aneurysms, or coarctation. We defined nonelective cases as unscheduled patients operated on within 24 hours after hospital admission. We detail statistical methods in the Supplemental Methods.

## Results

### Baseline and Operative Characteristics

We included 351 patients, mainly male (73%), with a median age of 61 years (interquartile range, 55-70 years) and operated on by 86 surgeons from 36 centers. Table 1 displays baseline demographics and patient characteristics between elective and nonelective index cases. Prevalence of type A dissections was significantly higher in nonelective cases (*P* < .0001), whereas reoperations (including patients who underwent urgent aortic repair as index surgery) were more frequent in elective cases (*P* < .0001). Nonelective patients also had a higher preoperative risk score evaluation (*P* < .0001) and were more likely to present with a preoperative stroke (*P* < .0001).

**Table 1:** Demographics and Preoperative and Intraoperative Data

Variable	Overall	Elective	Nonelective	*P*
No.	351	214	137	
Dissection Stanford classification				
Type A	161 (46)	54 (25)	107 (78)	**<.0001**
Type B	37 (11)	27 (13)	10 (7)	.07
Type non-A, non-B	15 (4)	11 (5)	4 (3)	.3
Nondissecting aneurysm	119 (34)	106 (50)	13 (9)	**<.0001**
Others	19 (5)	15 (7)	4 (3)	**.01**
Reoperation^a^	137 (37)	112 (52)	25 (18)	**<.0001**
Preoperative critical state^b^	41 (12)	1	40	**<.0001**
Log-EuroSCORE	13 (11-29)	19 (11-24)	29 (12-40)	**<.0001**
Age, y	61 (55-70)	62 (55-70)	59 (53-69)	.06
Sex, male	255 (73)	146 (68)	109 (79)	**.02**
Preoperative stroke	31 (9)	7 (3)	24 (18)	**<.0001**
Arteriopathy^c^	46 (13)	30 (14)	16 (12)	.6
Chronic obstructive pulmonary disease	35 (10)	25 (12)	10 (7)	.2
Creatinine >200 μmol/L	20 (6)	13 (6)	7 (5)	.7
Left ventricular ejection fraction				
>50%	285 (81)	178 (83)	107 (78)	.3
30%-50%	62 (18)	34 (16)	28 (20)	.3
<30%	4 (1)	2 (1)	2 (2)	.6

Categorical variables are presented as number (percentage). Continuous variables are presented as median with interquartile range (Q1-Q3). Boldface *P* values represent statistical significance. (EuroSCORE, European System for Cardiac Operative Risk Evaluation.)

a Reoperation: previous transsternal aortic or cardiac operation.

b Preoperative critical state: as assessed by the EuroSCORE.

c Arteriopathy: extra-aortic arterial disease.

### Early Outcomes

**Figure 1:**
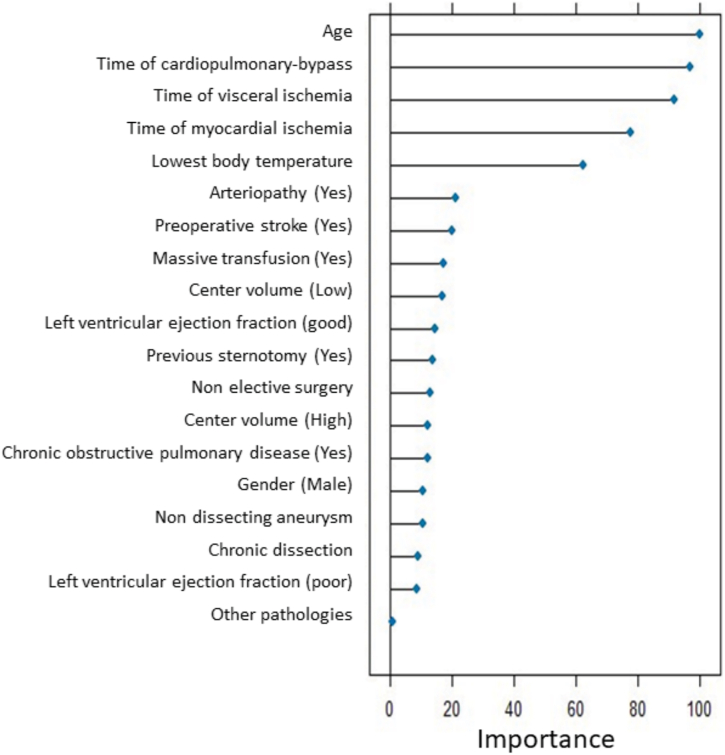
Classification random forest of early mortality according to preoperative and perioperative covariates. Accuracy = 0.85, out-of-bag estimate of error rate = 0.16.

Of 351 patients, 54 (15.4%) died postoperatively. Table 2 displays intraoperative data and outcomes between groups. FET in nonelective patients was more likely to be considered a 1-stage procedure (*P* = .01), to be performed with a short stent (*P* < 10^−4^), and to be associated with a longer time of selective cerebral anterograde perfusion (*P* = .005), with more aortic valve repair (*P* < 10^−4^). They presented postoperatively with more ischemic colitis (*P* = .001) and acute kidney injury requiring dialysis (*P* < 10^−4^). A funnel plot of the main end point among participating centers is presented in Supplemental Figure 2. Results of the classification random forest model (Figure 1) revealed that the most influential factors associated with in-hospital mortality were age, deep hypothermia, and each time of cardiopulmonary bypass, visceral ischemia, or myocardial ischemia. Less influential factors were preoperative arteriopathy, stroke, or critical state (as defined in the log-EuroSCORE risk scale); postoperative massive transfusion; previous sternotomy; and timing of surgery (elective or not) as well as center volume and indication for surgery. Accuracy of the random forest model was 0.85, and the out-of-bag estimate of error rate was 16%.

**Table 2:** Intraoperative Data and Outcomes

Variable	Overall	Elective	Nonelective	*P*
No.	351	214	137	
Operation time, min	362 (284-480)	376 (285-470)	360 (291-485)	.8
Cardiopulmonary bypass time, min	205 (165-262)	203 (165-252)	208 (164-270)	.5
Lowest body temperature, °C	25 (24-28)	25 (24-28)	25 (24-28)	.15
SCAP performed	273 (78)	163 (76)	110 (80)	.4
SCAP time, min	62 (45-85)	57 (42-80)	70 (50-98)	**.005**
Visceral ischemia time, min	49 (31-63)	50 (34-63)	48 (25-66)	.55
Cardiac ischemia time, min	115 (85-157)	117 (83-146)	115 (92-165)	.16
Long stent graft (15 cm)	80 (23)	65 (30)	15 (11)	**<.0001**
FET as single-stage procedure^a^	256 (73)	146 (68)	110 (80)	**.01**
Associated procedures				
Aortic valve replacement	62 (18)	41 (19)	21 (15)	.4
Aortic valve repair	62 (18)	15 (7)	47 (34)	**<.0001**
Aortic root replacement	63 (18)	34 (16)	29 (21)	.2
Coronary artery bypass grafting	27 (8)	17 (8)	10 (7)	.8
Other vascular surgery^b^	11 (3)	8 (4)	3 (2)	.4
In-hospital mortality	54 (15)	28 (13)	26 (19)	.1
Intraoperative death	6 (2)	2 (1)	4 (3)	.2
New permanent stroke	37 (11)	21 (10)	16 (12)	.6
SCI	20 (6)	10 (5)	10 (7)	.3
Permanent paraplegia	18 (5)	8 (4)	10 (7)	.1
Recurrent laryngeal nerve palsy	18 (5)	8 (4)	10 (7)	.1
Myocardial infarction	5 (1)	1 (0.5)	4 (3)	.6
Ischemic colitis	12 (3)	2 (1)	10 (7)	**.0014**
Severe low cardiac output syndrome^c^	6 (2)	3	3	.6
Massive blood transfusion^d^	34 (10)	18 (8)	16 (12)	.3
AKI requiring dialysis	60 (17)	18 (8)	42 (31)	**<.0001**
Reintervention during hospitalization				
For bleeding complication	36 (10)	23 (11)	13 (9)	.7
For additional vascular procedure	9 (3)	4 (2)	5 (4)	.3
Others (laparotomy)	13 (4)	4 (2)	9 (7)	**.02**
Secondary safety end point	124 (35)	74 (35)	50 (36)	.7

Categorical variables are presented as number (percentage). Continuous variables are presented as median with interquartile range (Q1-Q3). Boldface *P* values represent statistical significance.

AKI, acute kidney injury; FET, frozen elephant trunk; SCAP, selective cerebral anterograde perfusion; SCI, spinal cord injury.

a FET as single stage procedure: with no planned reintervention.

b Other vascular surgery: excluding direct or indirect reimplantation of supra-aortic vessels.

c Severe low cardiac output syndrome: low cardiac output syndrome responsible for severe organ dysfunction requiring extracorporeal membrane oxygenation or intra-aortic balloon pump.

d Massive blood transfusion: >10 packs of blood cells.

### Results From Follow-Up

**Figure 2:**
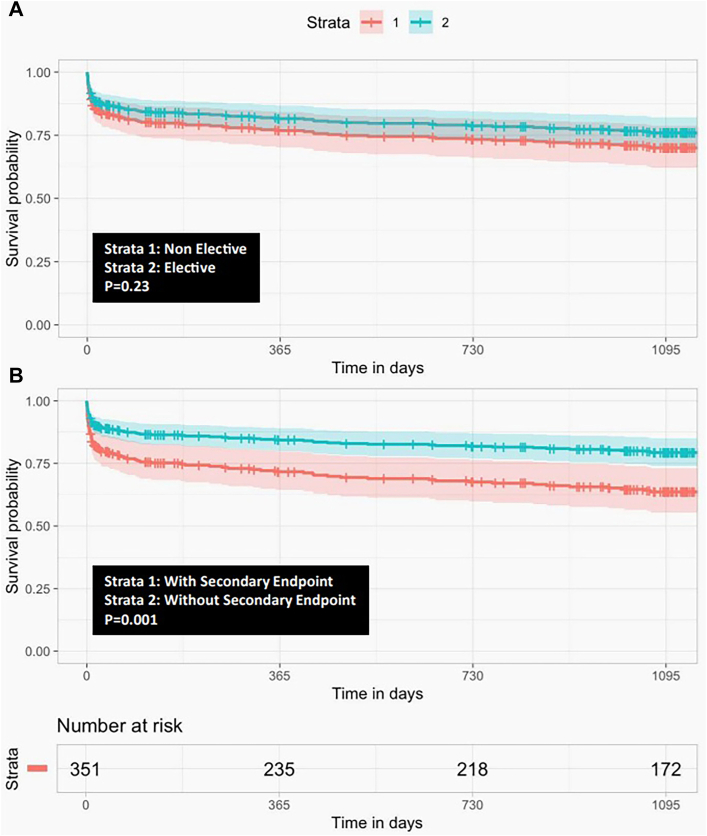
Cox proportional hazards model for age-adjusted crude survival (A) between studied groups and (B) according to second safety end point.

Median follow-up was 1149 (112-1323) days, during which 35 of 297 patients died. Overall, 3-year age-adjusted survival rates for elective and nonelective patients were 76% ± 6% and 70% ± 8%, respectively (*P* = .23). The secondary end point was strongly associated with in-hospital mortality (*P* = 10^−5^) and significantly lowered 3-year age-adjusted survival rates (hazard ratio = 2.34; *P* = .005; Figure 2). We display all details about aortic reoperations during follow-up and relative Kaplan-Meier estimates in the Supplemental Results, Supplemental Table, and Supplemental Figure 3.

## Comment

The EPI-Flex trial provides a comprehensive national overview of the early adoption of Thoraflex in aortic arch surgery in France. Real-world practices, which imply significant variability in patient selection and team expertise between different institutions, characterize this overview. We have previously published early results from the EPI-Flex trial focusing on nonelective cases.^2^ Herein, we completed the study transversally by including elective cases and longitudinally by extending observations to a complete 3-year follow-up. As a main result, we found comparable early and delayed survival between elective and nonelective cases with an overall in-hospital mortality at 15.4% and overall age-adjusted 3-year mortality at 73% ± 5%. We underline that the Epi-FLEX trial excluded neither elderly patients nor those in critical condition preoperatively, making it distinct from many other studies and complicating direct comparisons. Notably, regarding acute type A dissections, our results are competitive with those of The Society of Thoracic Surgeons database,^3^ which includes significantly fewer arch replacements. Nevertheless, the authors consider the Epi-FLEX trial an initial benchmark to be surpassed because continuous learning and refinement remain integral to the evolution of aortic teams. In particular, a better patient selection as well as expanding the use of various adjuncts could strengthen outcomes, as observed in high-volume and well-experienced centers.^4^

To better characterize operative risk, we also considered a secondary end point including any new neurologic insult, acute kidney injury requiring dialysis, massive bleeding, and unexpected repeated thoracic endovascular aortic repair or aortic surgery within 30 days. This end point was strongly related to in-hospital mortality (*P* = 10^−5^) and worsened midterm survival. It plainly justifies the ongoing research to minimize procedural risks of complications through procedure engineering, including careful preoperative assessment and computed tomography lecture, multiple arterial canulation sites, shifting proximally for collar anastomosis, early distal reperfusion, and close monitoring of hemostasis in the operating room.^5^, ^6^, ^7^

### Limitations

We designed this trial for safety assessment and not for a robust efficacy evaluation. Because the clinical status of critical patients biased functional assessment postoperatively, it is likely that the occurrence of the secondary end point might have been underestimated.

### Conclusion

We report on a wide comprehensive nationwide analysis of FET procedures performed in France using the Thoraflex in aortic arch replacement for various aortic diseases. The Epi-FLEX trial highlights that FET remains a complex and evolving procedure, just facilitated by the design of the Terumo Hybrid Plexus.
