# Supplementary material for: Frozen Elephant Trunk With Terumo Hybrid Plexus Prosthesis: A French Postmarket Longitudinal Study With Midterm Results
Source: Ann Thorac Surg Short Rep. 2025 Aug 28;4(1):6–11. doi: 10.1016/j.atssr.2025.07.024 (PMC13100794; doi:10.1016/j.atssr.2025.07.024)
Supplement: Supplementary Material [file mmc1.docx]

**SUPPLEMENTAL METHODS**

**Data Source:** Local surgeons were responsible for on-site patient registration as well as for accurate collection and recording of each patient data file, which implemented the EPI-Flex registry computer database through an electronic case report form. A data manager ensured quality of the database. Variables collected in this study included demographic data, determinants of the Log-Euroscore, indication and timing of surgery (elective versus urgent), time of cardiopulmonary bypass, circulatory arrest and myocardial ischemia, lowest body temperature, method of cerebral and spinal protection, complications, volume of transfused blood product, as well as data from follow-up (3 years) including mortality and reoperations.

**Statistical Analysis:** We used R version 4.3.0 (R Foundation for Statistical Computing) for all statistical analysis. In descriptive summaries, we expressed continuous variables as median with interquartile range (Q1-Q3) and categorical parameters as absolute numbers and percentages. We used one-way analysis of variance, two-tailed probability t-test or the Mann-Whitney U test, as appropriate, to compare continuous variables. We used two-way Pearson Chi-2 test or Fisher test whenever appropriate to compare categorical variables. We used the “Random Forest” package to establish a classification survival model and assess which individual pre and intra-operative factors influenced most 30-days mortality. We specified a formula using all features except the variable “Log-Euroscore” and SSE, set up a 10-fold cross-validation, and trained the model with 500 trees and 3 variables tried at each split. We used Cox proportional hazard models to analyze the relation between covariates and mortality. We compared crude survival between groups, **adjusting for age as a covariate by using** Cox-proportional hazards model. We used the Kaplan-Meier method to estimate time-to-reintervention rates among discharged patients with the “survival” package and we constructed cumulative actuarial rate of reoperation with Graph Pad Prism 9.0.2. We used Wald and Log-rank test to compare Cox-model and Kaplan-Meier estimates respectively. Survival estimates are expressed with a 95% confidence interval. We performed all analyses at a 2-sided significance level of 0.05. We made no adjustment for multiplicity tests.

**SUPPLEMENTAL RESULTS**

**Aortic reoperations:**

We defined unanticipated/unexpected reoperations as any open aortic, vascular or endovascular procedure including thoracic endovascular repair (TEVAR) performed during follow-up after a FET procedure, which had been designed as a single stage treatment by individual surgeons on site. Among discharged patients, the rate of any (p=0.05) and unexpected/unplanned reoperations (p=0.005) significantly increased after 3 years of follow-up in patients who initially underwent an elective FET (Supplemental Figure 3). Overall, 177 TEVAR or vascular/aortic reoperations, concerning 124 (42%) discharged patients were performed during follow-up **(Supplemental Table 1)**. Those iterative procedures were mainly TEVAR extension to cover distal aorta from zone Ishimaru 3 to nine, and were performed with an extension device (covert stent or RelayPro Stent-graft). Concerning early device related complications, we could identify three Type IB endoleaks related to an inadequate fixation of the covered stent at the distal end of the graft and leading to an incomplete seal requiring an urgent TEVAR due to a fast-growing aortic diameter because of its high-pressure nature. One patient presented a stent under-deployment requiring ballooning. Two patients presented FET thrombus, one resolving spontaneously under anticoagulant therapy, the other requiring TEVAR in order to apply the thrombus on aortic wall. One patient treated for acute dissection presented an early aortic rupture suggesting a FET malposition into the false lumen. Concerning late device related complications, we report on two device infections and eight Type II endoleaks considering that 26 patients had a planned distal seal in the FET as part of a remodeling induced with STABILIZE device (aortic dissections) or as part of the treatment of thoraco-abdominal aneurysms.

**SUPPLEMENTAL ACKNOWLEGMENT**

**List of non-authors contributors** (database manager* and participating investigators): ABI AKAR Ramzi; ABOULIATIM Issam; ACHOUH Paul; ALKHODER Soleiman; ALUDAAT Chadi; AZARNOUSH Kasra; BARANDON Laurent; BARREDA Eleodoro; BEL Alain; BERGOEND Eric; BERTHOUMIEU Pierre; BERTRAM Maxime; BILAND Guillaume; BONNET Nicolas; BOUCHOT Olivier; BOURGUIGNON Thierry; BRAUNBERGER Eric; CAMILLERI Lionel; CARMI Doron; CAUS Thierry; CHAKFE Nabil; CHAVANIS Nicolas; CHICHE Laurent; CHOCRON Sydney; COLLART Frédéric; COUETIL Jean-Paul; CRON Christophe; CURTIL Alain; DOISY Vincent; D'OSTREVY Nicolas; DU PUY MONTBRUN Leonora; DUBOIS Gilbert; FARAMAND Patrick; FARHAT Fadi; FAVRE Jean-Pierre; FOLLIGUET Thierry; FOUQUET Olivier; FUZELLIER Jean-François; GANDET Thomas; GEORG Yannick; GERELLI Sébastien; GRISOLI Dominique; GRUNENWALD Etienne; GUHAIRE Julien; JOUAN Jérome; KOSKAS Fabien; LANSAC Emmanuel; LAURENT Nicolas; LEPRINCE Pascal; LOARDI Paula; MALAPERT Ghislain; MARCHEIX Bertrand; MAUREIRA Miguel, OSES Pierre; PELTAN Julien; PERNOT Mathieu; PICARD Carl*; PINELLI Samuel; PORCU Paolo; PORTERIE Jean; PORTOCARRERO Erick; RADU Costin; RAFFOUL Richard; ROUSSEL Jean-Philippe; ROUVIERE Philippe; RUBIN Sylvain; RUGGIERI Vito Giovanni; SENAGE Thomas; THAVEAU Fabien; VAN HUYSE Fabrice; VINCENTELLI André; VOLA Marco; ZANNIS Konstantinos.

**LEGEND TO SUPPLEMENTAL FIGURES:**

Supplemental Figure 1: Chart-flow of patients’ inclusions.

Supplemental Figure 2: Funnel plot of observed in-hospital mortality among participating centers.

Supplemental Figure 3: Kaplan-Meier estimates of freedom from all (any) reoperation (a) and from unplanned/unexpected reoperation (b) among discharged patients between studied groups.
