# Supplementary material for: Frozen Elephant Trunk With Terumo Hybrid Plexus Prosthesis: A French Postmarket Longitudinal Study With Midterm Results
Source: Ann Thorac Surg Short Rep. 2025 Aug 28;4(1):6–11. doi: 10.1016/j.atssr.2025.07.024 (PMC13100794; doi:10.1016/j.atssr.2025.07.024)
Supplement: Supplementary Table 1 [file mmc2.docx]

|  | Planned/Expected | Unplanned/Unexpected | p |
| --- | --- | --- | --- |
| N (patients) | 54 | 70 |  |
| n (procedures) | 86 | 91 |  |
| FET (indication)  *Elective (%)*  *Non Elective (%)*  Type A dissection (%)  Type B dissection  Type non-A non-B dissection  Non dissecting aneurysm  Other pathologies | *40 (74)*  *14 (26)*  26 (48)  5 (9)  1 (2)  20 (37)  2 (4) | *53 (75)*  *17 (25)*  28 (40)  12 (17)  4 (6)  22 (31)  4 (6) | 0.83  0.2  0.47  0.41  0.1  0.6 |
| Time from FET to first reoperation | 60 (16-181) | 307 (104-892) | **0.001** |
| Endovascular (%)  Open surgery (%) | 70 (81)  16 (19) | 64 (70)  27 (30) | 0.14 |
| In-hospital mortality following reoperation | 5 (9.3%) | 3 (4.3%) | 0.26 |

**Supplemental Table 1:** Details on vascular/aortic reoperation as defined (planned versus unplanned) among discharged patients.
