# Supplementary figures and images for: Frozen Elephant Trunk With Terumo Hybrid Plexus Prosthesis: A French Postmarket Longitudinal Study With Midterm Results
Source: Ann Thorac Surg Short Rep. 2025 Aug 28;4(1):6–11. doi: 10.1016/j.atssr.2025.07.024 (PMC13100794; doi:10.1016/j.atssr.2025.07.024)

## Supplemental Figure 1

FET: Frozen Elephant Trunk  
THP: Thoraflex Hybrid Prosthesis

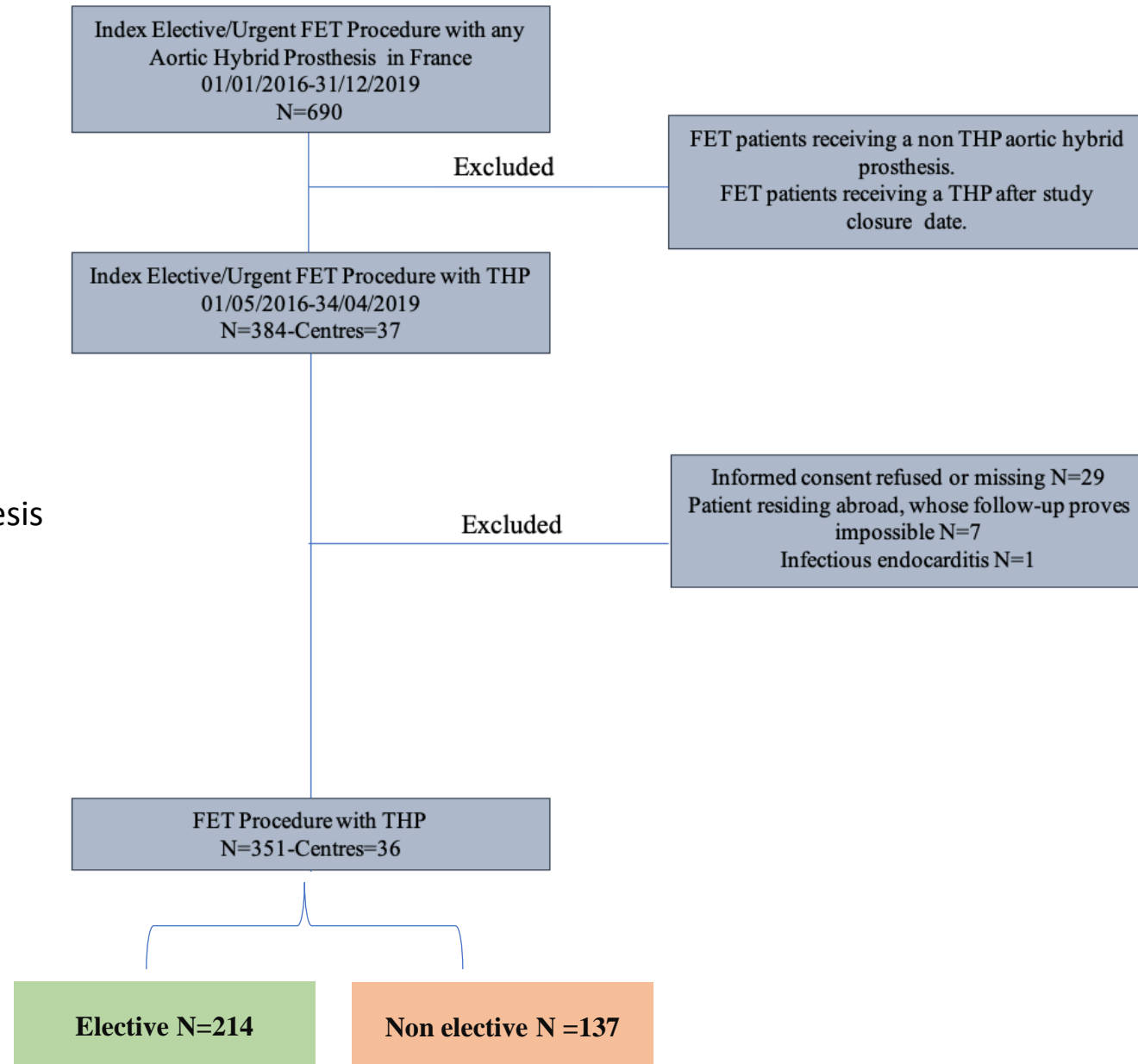

Supplement: Supplementary Figure 1 [file mmc3.pdf]

Supplemental Figure 2

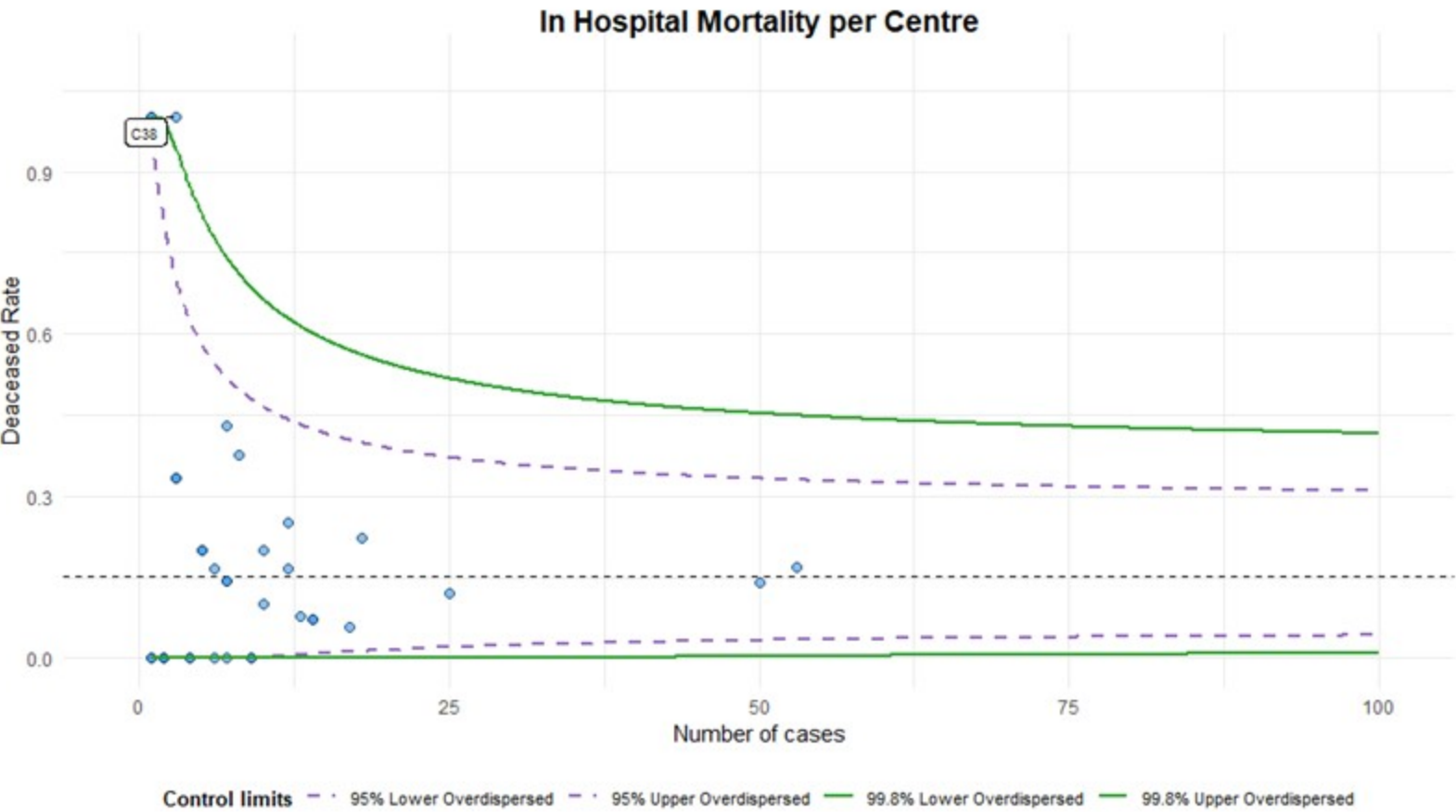

Supplement: Supplementary Figure 2 [file mmc4.pdf]
