# Supplementary material for: Frozen Elephant Trunk With Terumo Hybrid Plexus Prosthesis: A French Postmarket Longitudinal Study With Midterm Results
Source: Ann Thorac Surg Short Rep. 2025 Aug 28;4(1):6–11. doi: 10.1016/j.atssr.2025.07.024 (PMC13100794; doi:10.1016/j.atssr.2025.07.024)
Supplement: Supplementary Figure 3 [file mmc5.pdf]

Supplemental Figure 3 a

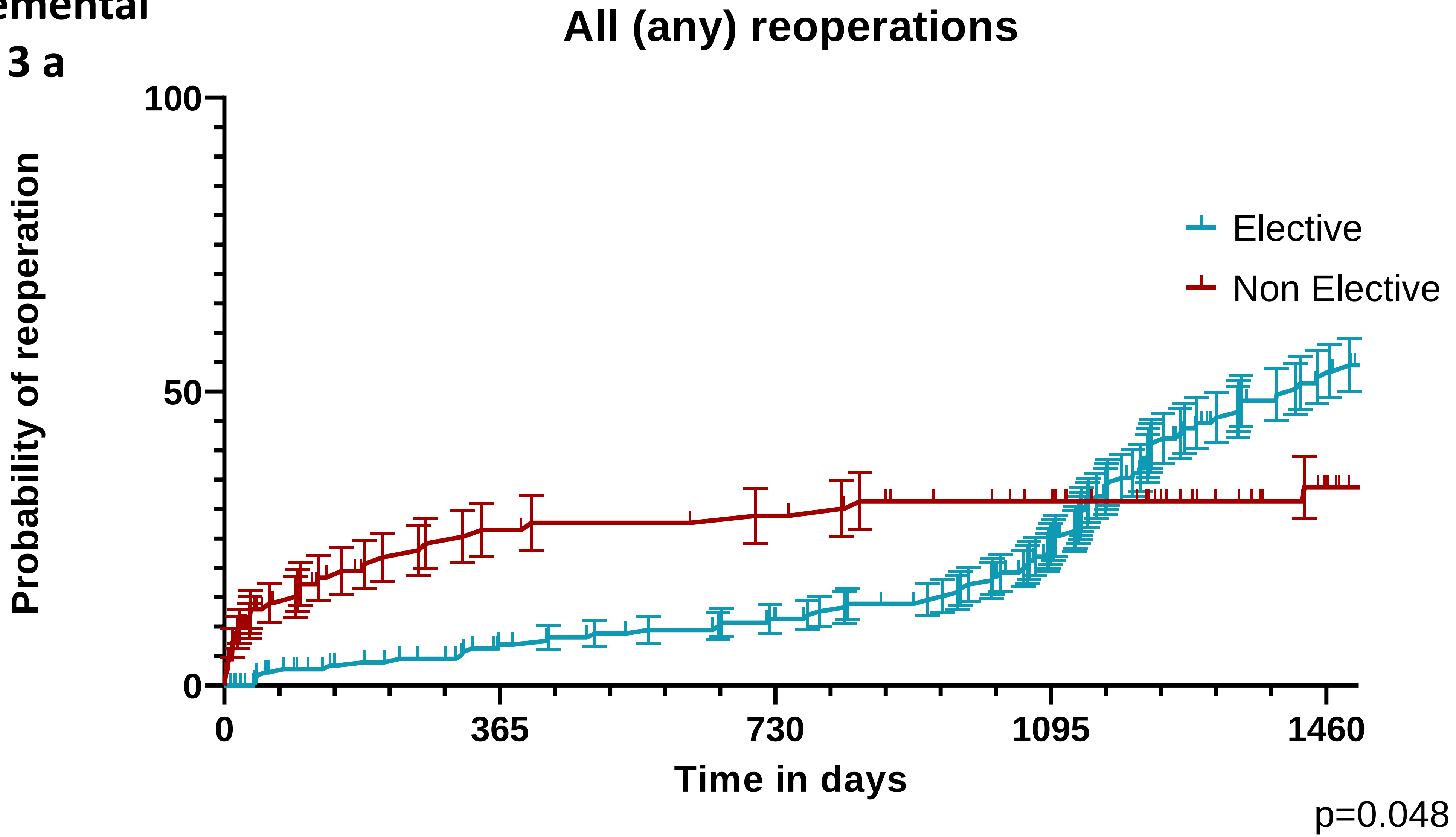

**Elective**	**186**	**153**	**139**	**107**	**47**
Non Elective	111	64	60	50	26

Supplemental Figure 3 b

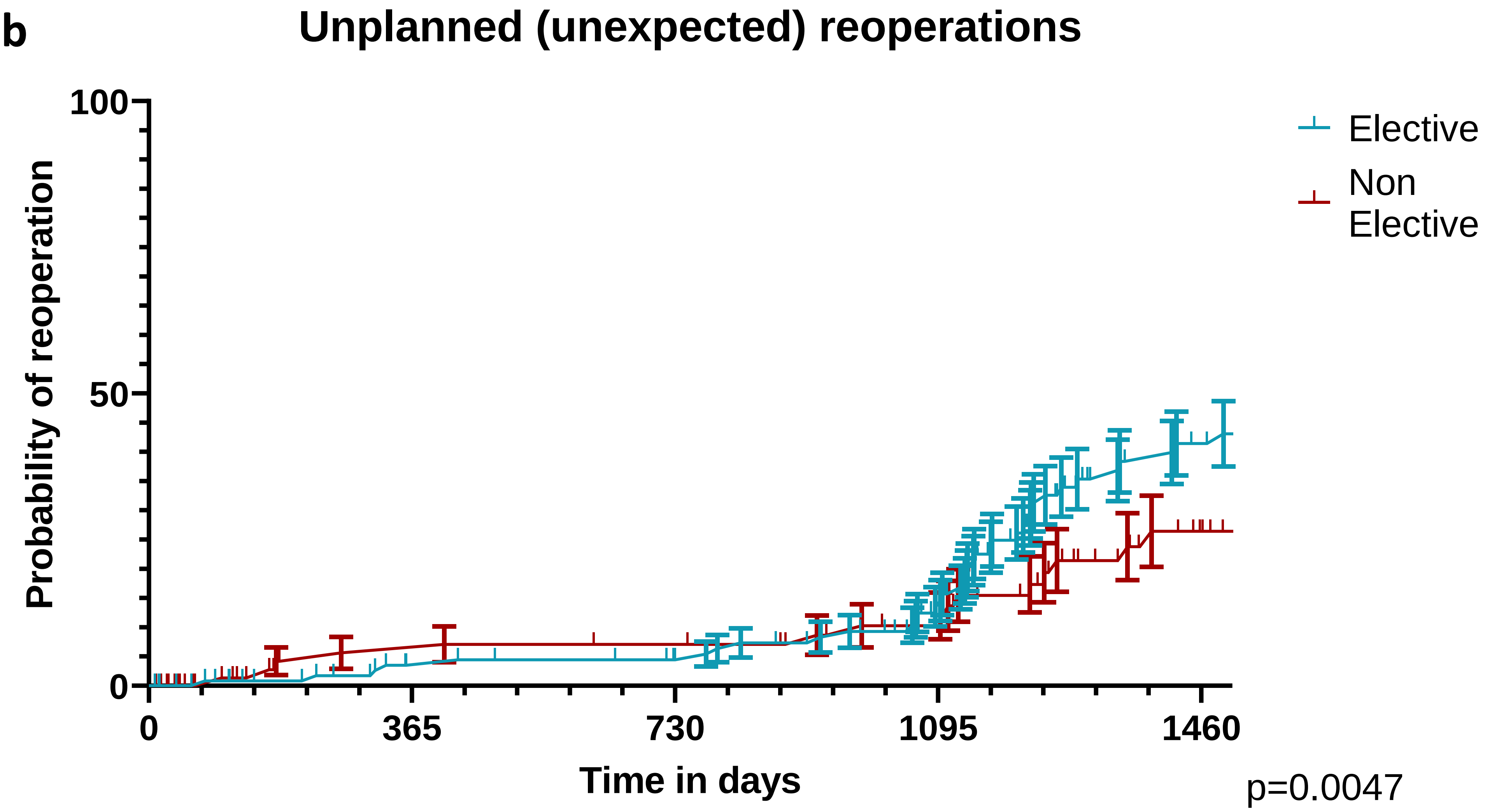

**Elective**	**125**	**106**	**100**	**80**	**38**
Non Elective	89	66	63	55	26
